# Deep learning as a tool for increased accuracy and efficiency of histopathological diagnosis

**DOI:** 10.1038/srep26286

**Published:** 2016-05-23

**Authors:** Geert Litjens, Clara I. Sánchez, Nadya Timofeeva, Meyke Hermsen, Iris Nagtegaal, Iringo Kovacs, Christina Hulsbergen - van de Kaa, Peter Bult, Bram van Ginneken, Jeroen van der Laak

**Affiliations:** 1 Department of Pathology, Radboud University Medical Center, Nijmegen, The Netherlands; 2 Department of Radiology and Nuclear Medicine, Radboud University Medical Center, Nijmegen, The Netherlands; 3 Department of Pathology, Amphia Breda Medical Center, The Netherlands

## Abstract

Pathologists face a substantial increase in workload and complexity of histopathologic cancer diagnosis due to the advent of personalized medicine. Therefore, diagnostic protocols have to focus equally on efficiency and accuracy. In this paper we introduce ‘deep learning’ as a technique to improve the objectivity and efficiency of histopathologic slide analysis. Through two examples, prostate cancer identification in biopsy specimens and breast cancer metastasis detection in sentinel lymph nodes, we show the potential of this new methodology to reduce the workload for pathologists, while at the same time increasing objectivity of diagnoses. We found that all slides containing prostate cancer and micro- and macro-metastases of breast cancer could be identified automatically while 30–40% of the slides containing benign and normal tissue could be excluded without the use of any additional immunohistochemical markers or human intervention. We conclude that ‘deep learning’ holds great promise to improve the efficacy of prostate cancer diagnosis and breast cancer staging.

Microscopic analysis of hematoxylin and eosin (H&E) stained sections has been the basis for cancer diagnosis and grading for the past century^1^. Protocols for the complete workup of biopsies or resected tissue specimens, including microscopic analysis, exist for many of the most common cancer types (e.g. lung, breast, prostate). Use of these protocols has led to strong prognostic and widely used grading strategies (e.g. the Gleason grading system)^2^.

Due to the rise in cancer incidence and patient-specific treatment options, diagnosis and grading of cancer has become increasingly complex. Pathologists nowadays have to go over a large number of slides, often including additional immunohistochemical stains, to come to a complete diagnosis. Moreover, there is an increase in the amount of quantitative parameters pathologists have to extract for commonly used grading systems (e.g. lengths, surface areas, mitotic counts)^3^. Due to these difficulties, analysis protocols have been adapted and fine-tuned to offer the best balance between prognostic power and feasibility in daily clinical routine^4^.

The recent introduction of whole-slide scanning systems offers an opportunity to quantify and improve histopathologic procedures. These systems digitize glass slides with stained tissue sections at high resolution. Digital whole slide images (WSI) allow the application of image analysis techniques to aid pathologists in the examination and quantification of slides^5^. One such technique which has gained prominence in the last five years in other fields is ‘deep learning’^6^. While ‘deep learning’ cannot be considered a single technique, it can roughly be described as the application of multi-layered artificial neural networks to a wide range of problems, from speech recognition to image analysis. In recent years, ‘deep learning’ techniques have quickly become the state of the art in computer vision. A specific neural network subtype (convolutional neural networks; CNN^7^,^8^ has become the de facto standard in image recognition and is approaching human performance in a number of tasks^6^. These systems function by learning relevant features directly from huge image databases (typically millions of images). This is in contrast to more traditional pattern recognition techniques, which strongly rely on manually crafted quantitative feature extractors.

In spite of these huge successes, ‘deep learning’ techniques have not yet made a big impact on the field of medical imaging. One of the main reasons is that for the traditional imaging based specialties (e.g. radiology) the large numbers of images that are needed to train complex ‘deep learning’ systems are not readily available. In digital histopathology this is easier: one WSI typically contains trillions of pixels from which hundreds of examples of cancerous glands (in the case of prostate or breast cancer) can be extracted.

Some initial work has been published over the last five years discussing the application of ‘deep learning’ techniques to microscopic and histopathologic images. Ciresan *et al*. were the first to apply convolutional neural networks to the task of mitosis counting for primary breast cancer grading^9^. Furthermore, in a different publication, they showed the applicability of patch-driven convolutional neural networks to segmentation tasks^10^. Wang *et al*. later expanded the work on mitosis detection by combining hand-crafted features and convolutional neural networks^11^. Other applications of convolutional networks include primary breast cancer detection^12^, glioma grading^13^ and epithelium and stroma segmentation^14^. Last, Su *et al*. used another ‘deep learning’ technique, called stacked denoising auto-encoders to perform cell detection and segmentation in lung cancer and brain tumors^15^.

This study investigates the general applicability of CNNs to improve the efficiency of cancer diagnosis in H&E images by applying it to two novel tasks: the detection of prostate cancer in biopsy specimens and the detection of breast cancer metastases in resected sentinel lymph nodes.

The number of prostate biopsy sections has strongly increased in the past decades due to the advent of prostate specific antigen (PSA) testing^16^. Because of the nature of the standard biopsy procedure (eight to twelve random biopsies under ultrasound-guidance), each procedure results in several slides. The majority of these slides typically do not contain cancer. The histopathological analysis could be streamlined significantly if these normal slides could automatically be excluded without expelling any slides containing cancer. We collected consecutive single-center biopsy specimens of 254 patients who underwent MR-guided biopsy procedures for prostate cancer at our institution. These specimens were prepared according to standard histopathologic protocol and subsequently digitized using an Olympus VS120-S5 system (Olympus, Tokyo, Japan).

The sentinel lymph node procedure is well known for its tedious inspection protocol^4^. Several sections of the lymph node have to be investigated for micro-metastases (0.2–2 mm) and macro-metastases (>2 mm). Furthermore, around 60–70% of the sentinel lymph nodes do not contain any metastases^17^. In this paper we focus on the sentinel lymph node procedure for breast cancer with the aim of identifying slides which do not contain micro- or macro-metastases. Also, we tried to identify the correct location of the metastases within a specific slide. In total 271 patients were included from our institution. Specimens were prepared according to the standard histopathologic protocol and subsequently digitized using a 3DHistech Pannoramic 250 Flash II scanner (3DHistech, Budapest, Hungary).

After digitization of the H&E-stained slides cancer and metastases were manually delineated using a computer mouse by a resident of pathology (I.K., prostate cancer experiment) and a lab technician (M.H., sentinel lymph node experiment), under the supervision of experienced pathologists (C. A. H. K., P. B.). From these annotated areas small prototype image regions (‘patches’) were extracted to train CNNs to detect cancer areas in validation data sets (schematic overview in Fig. 1). These validation data sets were used to optimize the network parameters. After training, the CNN was converted to a fully convolutional network which gave per-pixel predictions on the presence of cancer and metastases in separate, not previously used, test data sets. For prostate cancer detection the CNNs were evaluated on a per-slide level using receiver-operator curve (ROC)-analysis. We also investigated how well the system could exclude slides without cancer from further diagnostic processing. For the sentinel lymph node procedure, we assessed how well the system was capable of identifying individual micro- and macro-metastases using free-response ROC (FROC) analysis and if it is capable of excluding slides which do not contain any metastases using ROC analysis.

## Results

### Subjects

#### Prostate cancer

From the initial set of 254 patients, eleven were excluded because the glass slides were not available. Four were excluded because no biopsy was taken during the procedure and one was excluded as the tissue sample was too small for pathologic analysis. Out of the remaining 238, we randomly selected 225 glass slides for digitization, of which 100 were assigned to the training set, 50 to the validation set and 75 to the test set. The training set sampling was stratified such that a near-50/50-distribution between slides containing cancer and slides not containing cancer was obtained. All slides were successfully digitized and annotated. Further details on the selected slides can be found in Table 1.

#### Breast cancer sentinel lymph nodes

Data collection for the sentinel lymph node experiments was performed in two batches. The first batch was obtained by including 173 slides from the case files of an experienced breast pathologist (P.B). These initial slides were split into a training (98), validation (33) and test (42) set. These slides were subsequently digitized and every metastasis was annotated. To make sure our results were not biased to a single pathologist’s case selection, we acquired a second set of data by including all the consecutive sentinel lymph node cases for breast cancer from October 2014 to April 2015, resulting in an additional 98 whole-slide images. For the second batch no on-slide annotations were available, only the per-case outcome (presence of macro- and/or micro-metastases and isolated tumor cells (ITC)). Further details on the included cases can be found in Table 2. Of cases with only ITC, 22 out of 24 had additional immunohistochemistry ordered by the pathologist.

### Prostate cancer detection

**Figure 2:**
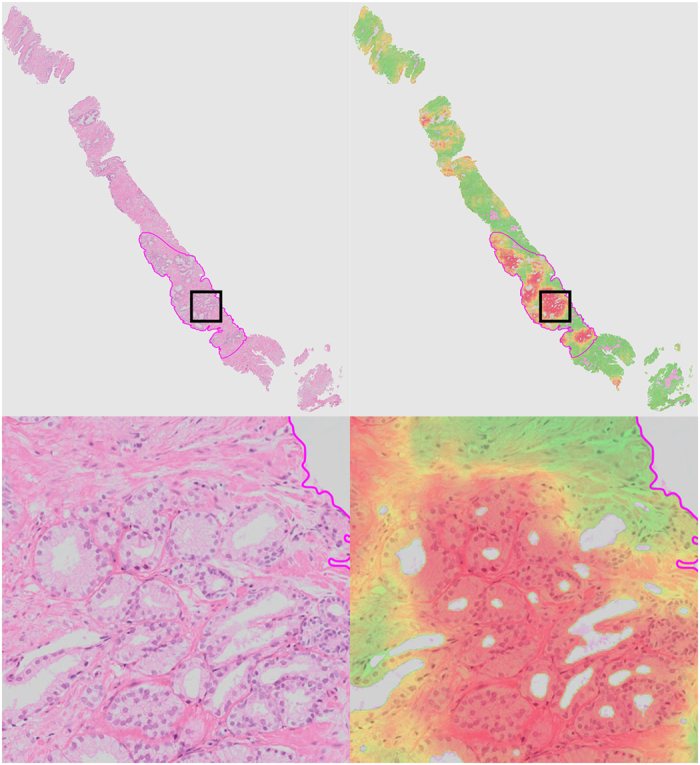
Representative example of a whole slide prostate biopsy specimens with 30% cancer. The top row shows the complete field of view, the bottom row a close up (close-up area indicated by the square rectangle). The second column shows the cancer likelihood map as an overlay on the original image. Red indicates a high likelihood of cancer, whereas transparent/green indicates a low likelihood.

A cancer likelihood map (CLM), the output of the CNN indicating cancer likelihood per pixel, for a representative WSI from the test set with cancer covering 30% of the tissue area is shown in Fig. 2. The cancerous glands indicated by the pathologist’s outline (in magenta) are correctly identified with high likelihood. The stroma within the annotation areas is correctly identified as a low cancer likelihood region (in green, most easily identifiable in the high-resolution sub-images).

**Figure 3:**
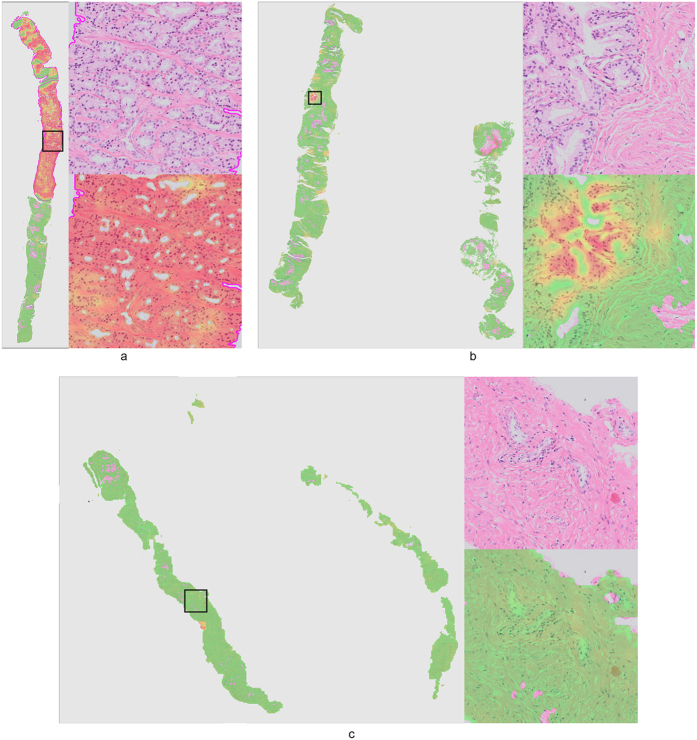
Three representative examples of a whole slide prostate biopsy specimen. Each example (**a**–**c**) shows the complete field of view with the cancer likelihood map as an overlay. Red indicates a high likelihood of cancer, whereas transparent/green indicates a low likelihood. Example (**a**) contains around 40% cancer (indicated by the magenta outline), examples (**b**,**c**) do not contain cancer. Close-up sub-images are shown for the areas indicated by black square. For example (**b**) we choose to highlight a small false positive area caused by tissue deformation at the edges of the biopsy.

Several other examples are presented in Fig. 3. In Fig. 3b, we show a high-resolution sub-image of a false positive region. Due to cutting and histopathologic processing, tissue at the edges of the biopsy specimens often deforms and tears, resulting in abnormal appearance. If we examine this area closely, we can see that the false positive glands indeed show some features which are comparable to those of cancer (e.g. fusing glands, irregular shape). In general, we can clearly see a distinct separation between malignant (Figs 2 and 3a) and benign biopsy specimens (Fig. 3b,c) based on the CLMs.

**Figure 4:**
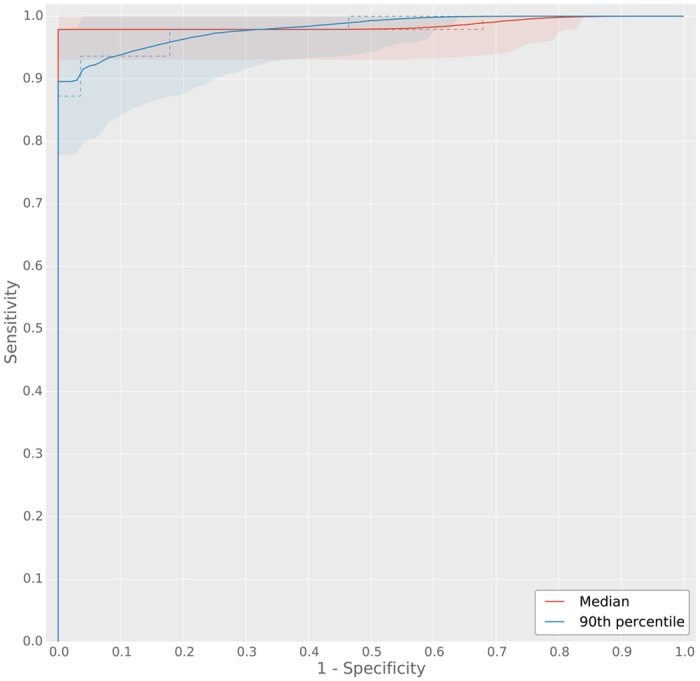
Receiver operating characteristic (ROC) curves for the cumulative histogram analysis in the whole-slide prostate biopsy experiment. Two cumulative histogram parameters were used to obtain ROC curves, the median and 90^th^-percentile of the cumulative histogram of the whole slide images. The median ROC curve has a higher area under the curve (AUC), however, the 90^th^-percentile ROC curve shows higher specificity at high sensitivity. Solid lines indicate the mean bootstrapped ROC curve, the shaded areas indicate the 95^th^-percentile confidence intervals and the dashed line indicates the raw ROC curve.

Quantitatively, the result of performing histogram analysis on the CLMs can be best represented using ROC analysis. In Fig. 4 we present the ROC curves for both median and 90^th^-percentile analysis of the cumulative histogram of the CLM over the independent test set. Indicated with the dashed lines are the raw ROC curves, the solid line and the shaded areas represent the mean bootstrapped ROC curve and the 95^th^-percentiles. Bootstrap is a statistical technique which enables estimation of confidence intervals by repeated resampling of a representative population^18^. The average bootstrapped area under the ROC curve (AUC) for the median analysis was 0.99 (0.95–1.0) and 0.98 (0.94–0.99) for the 90^th^-percentile analysis. However, the 90^th^-percentile analysis has higher specificity at a sensitivity level of 0.999 (0.32, 95%-CI: 0.29–0.97) compared to median analysis (0.17, 95%-CI: 0.15–1.0).

### Identification of breast cancer metastases in sentinel lymph nodes

**Figure 5:**
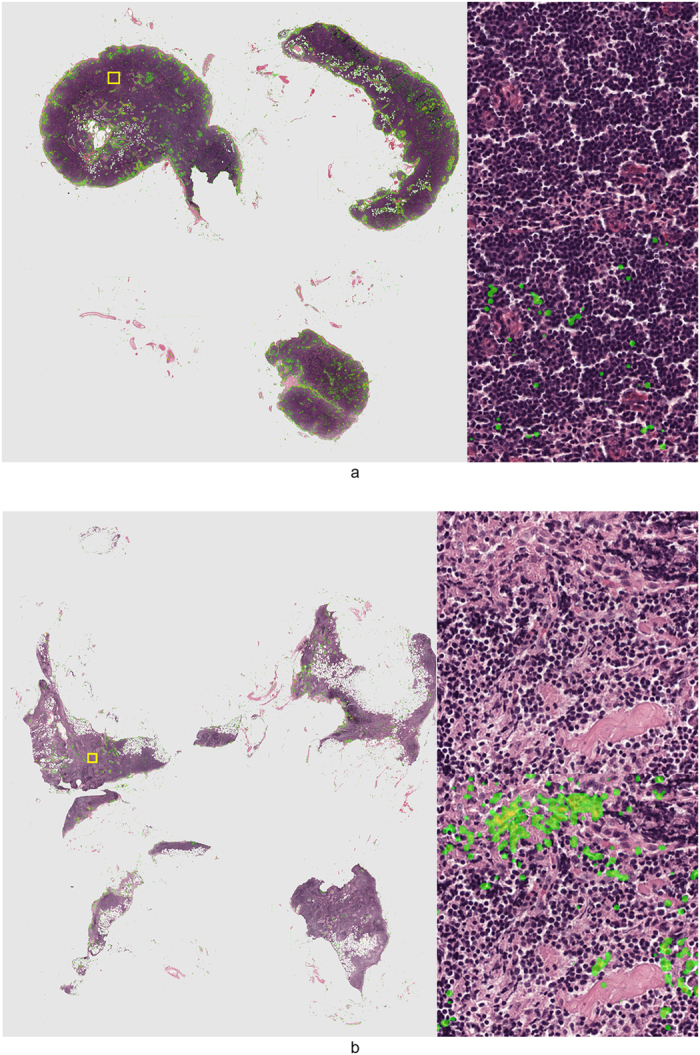
Representative examples of normal lymph nodes from the consecutive set. Metastases likelihood maps are overlaid on the original H&E image. Transparent/green means a low likelihood, whereas red indicates a high likelihood of metastasis. On the right side of the whole slide images the areas indicated by the yellow squares are shown at full-resolution.

**Figure 6:**
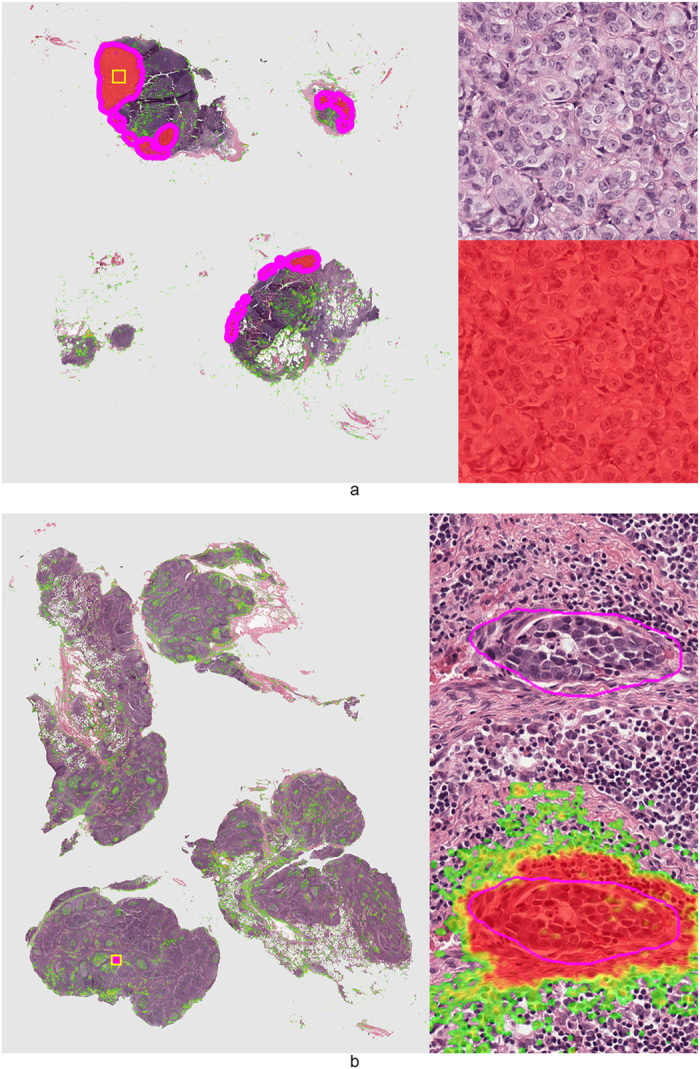
Representative examples of lymph nodes with macro-metastases (top image) and a single micro-metastasis (bottom image) from the test set. Metastases likelihood maps are overlaid on the original H&E image. Transparent/green means a low likelihood, whereas red indicates a high likelihood of metastasis. Magenta contours indicate the ground truth annotation. On the right side of the whole slide images the areas indicated by the yellow squares are shown at full-resolution.

Representative examples of sentinel lymph node specimens are presented in Fig. 5 (without metastases) and Fig. 6 (with metastases). The metastases are correctly detected with very high likelihood (red color). Areas containing only lymphocytes are mostly transparent (likelihood close to zero), whereas areas containing histiocytes or mixtures of histiocytes and lymphocytes are transparent – green (low likelihoods). Distinction between histiocyte–rich regions and metastases is also a well-known difficulty for residents in pathology.

**Figure 7:**
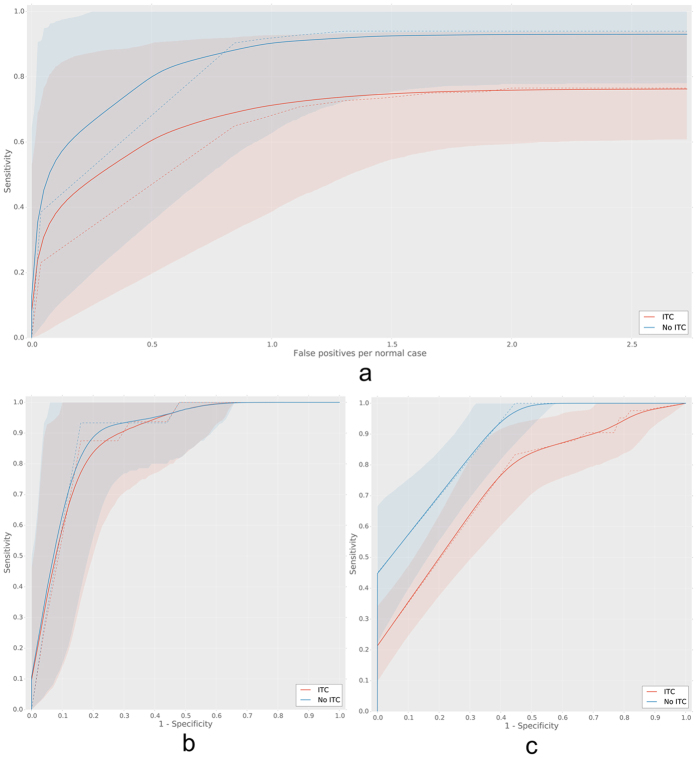
Bootstrapped FROC and ROC curves for the lymph node experiments. Subfigure (**a**) contains the FROC curve on the test set, (**b**) contains the ROC curve on the test set and (**c**) contains the ROC curve on the consecutive data. Curves for both including (red) and excluding isolated tumor cells (ITCS (blue) from the analysis are shown. Solid lines indicate the mean bootstrapped ROC curve, the shaded areas indicate the 95th-percentile confidence intervals and the dashed line indicates the raw ROC curve.

Quantitatively, results were analyzed in two ways. FROC analysis was used to assess localization accuracy, whereas ROC analysis was used to assess performance at the slide level. FROC analysis was only performed on the test set, as annotations are required to assess localization accuracy. The FROC and ROC curves are shown in Fig. 7. Results of the FROC and ROC analyses are also summarized in Table 3. At the expense of one or two false positive detections per tumor-negative image 90% or 93% of all individual micro- and macro-metastases could be identified, respectively. If we also include all isolated tumor cell (ITC) instances, 71% was found at the expensive of one false positive detection per tumor-negative image and 74% was found at the expense of two false positive detections per image.

The ROC analysis shows that in both the test and consecutive data sets, an area under the ROC curve of close to 0.90 can be obtained on the slide level when discriminating slides without from slides with micro- and macro-metastases. Furthermore, at 0.999 sensitivity up to 0.44 specificity could be obtained in the consecutive set. When also including slides only containing ITC, performance drops to an area under the ROC curve of 0.74 and 0.02 specificity at 0.999 sensitivity.

## Discussion

Although deep learning is an active research field, the application of deep learning to histopathology is relatively new. Most already published work has focused on the detection of mitotic figures^9^,^11^ or identification and segmentation of individual cells^15^,^19^. One paper used a convolutional auto-encoder to segment basal-cell carcinoma in H&E-images of breast cancer^20^.However, this model is only evaluated on images from pre-selected regions of interest and not on whole slides, making it difficult to assess its practical value.

The two papers most closely related to our work have focused on different entities. Cruz-Roa *et al*. used a CNN to detect and segment primary breast cancer^12^ and Ertosun *et al*. investigated the grading of gliomas^13^. We explored the applicability of CNNs to digitized histopathology through two different experiments: prostate cancer detection in H&E-stained biopsy specimens and identification of metastases in sentinel lymph nodes obtained from breast cancer patients. In contrast to these two papers, which perform patch-by-patch classification, we use fully convolutional networks to obtain per-pixel cancer likelihood maps and segmentations in whole-slide images. Furthermore, we are the first to report slide-level accuracies for cancer detection.

In both experiments we were able to successfully train convolutional neural networks, although the amount of case data was less than what is generally typical in ‘deep learning’ experiments. The fact that we performed extensive data augmentation and boosting in combination with the relatively limited domain (i.e. H&E-stained histopathologic images compared to natural images) made this possible.

In both applications we investigated whether it was possible to identify slides not containing disease without overlooking any slides containing disease. In the prostate cancer slides, up to 32% of the slides not containing disease could be excluded. For the sentinel lymph nodes, specificity was even higher at 44%, without missing any slide containing micro- or macro-metastases. This indicates that substantial gains in efficiency are possible by using CNNs to exclude tumor-negative slides from further human analysis.

Next to the performance of the CNN at high sensitivity, area under the ROC curve was also high in both cases, with an AUC of 0.99 for the prostate cancer experiment (median analysis) and 0.88 for the sentinel lymph node experiment (consecutive set). Furthermore, localization accuracy was high for micro- and macro-metastases in the sentinel lymph node experiment (90% sensitivity at 1 false positive per normal image).

There are some limitations to the application of the CNNs, especially for the sentinel lymph nodes. Although the accuracy of detecting micro- and macro-metastases is high, adding the requirement of having to identify all clusters of isolated tumor cells lowers performance significantly (0.74 AUC for the consecutive set). However, the importance of ITCs is debated. Some have found no prognostic implication of ITCs^4^,^21^ at all or when the ITCs are visible through immunohistochemistry only^15^,^16^. Others did find ITCs having a negative prognostic impact, albeit effect sizes differ^22^,^23^,^24^. However, for the clinical application of CNNs this is of limited importance. If the application of the CNNs can detect the micro- and macro-metastases with high accuracy, and we have shown this, the ITCs can be detected by immunohistochemistry, without having a pathologist looking at the H&E stained slides. In The Netherlands, according to the national guideline for breast cancer, immunohistochemistry is mandatory when no tumor is found in the H&E-stained slides.

In the prostate cancer experiment, some detection errors (i.e. false positive detections) still occur at the boundaries of the tissue, mostly due to tearing and tissue deformation. These are expected artifacts that occur during histopathologic processing, and ideally our CNN would be robust to this. However, due to the fact that these artifacts can have a wide range of appearances and only occur sporadically, this is not yet the case with the size of the training set used in this study. For the slide level analysis, these spurious detections are not problematic; they occur equally in slides containing and not containing cancer, making their separation still possible.

One further limitation is the fact that we only investigated data from a single center. Although we included data from distinctly different tissue types and used digitization equipment from two different vendors, it is important that these results are confirmed in future, multi-center studies.

As far as the authors are aware, this is the first paper describing the general applicability of a ‘deep learning’ technique to the diagnostic analysis of whole slide images of sentinel lymph nodes and prostate biopsies. We have shown that this technique is potentially highly suitable to improve the efficiency of the diagnostic process in histopathology. This could in turn lead to adapted protocols, where pathologists perform a more detailed analysis on the difficult samples, as the easy samples are already handled by a computer system.

Although we specifically looked at clinical diagnosis in this study, the potential of these ‘deep learning’ techniques reaches further. They could also be used to quickly analyze huge clinical trial databases to extract relevant cases, or automatically annotate areas of disease to allow fast quantification (e.g. area, diameter). Furthermore, the technique is not limited to H&E-stained images and could readily be applied to immunohistochemistry, which might be of interest when researching the efficacy of drugs or the expression of genes. Both are worthwhile avenues for future research.

## Methods

### Materials

For all patients in this study the institutional review board waived the need for informed consent.

#### Prostate cancer

A search was performed on our institution’s PACS system to identify all patients who underwent MR-guided biopsy of the prostate after an initial multi-parametric MRI suspicious for cancer in 2012. This resulted in a total of 254 patients who were initially included in this study. After assessing suitability for inclusion, patients were randomly assigned to one of three sets: training, validation and test.

The biopsy specimens were previously stained using standard protocols for H&E-staining used in routine clinical care, after which the specimens were analyzed and reported on by an experienced pathologist as part of routine diagnostics. For this study these glass slides were obtained from the archive of the Department of Pathology for subsequent digitization.

#### Sentinel lymph node

The sentinel lymph node case files of an experienced pathologist (P.B.) were used to identify subjects to include in the initial training set. The sentinel node specimens were previously stained using standard protocols for H&E-staining used in routine clinical care, after which the specimens were analyzed and reported by the experienced pathologist as part of routine diagnostics.

To make sure our results were not biased incidentally by only evaluating on the cases obtained from one pathologist’s case file, we included all sentinel lymph node subjects from October 2014–April 2015 as an extra testing set.

One H&E-stained slide per subject was subsequently obtained from the archive at the Department of Pathology for digitization. Slides were selected such that they contained the largest area of metastases, if metastases were present.

### Digitization and annotation

#### Prostate cancer

Prostate cancer slides were digitized using an Olympus VS120-S5 slide scanning system. Slides were digitized using a 40× objective (resultant pixel resolution of 0.16 microns). After digitization the digital slides were annotated for cancer using an in-house developed freehand drawing tool. Annotation was performed by a resident of pathology (I.K.) under the supervision of an experienced pathologist (C.H.-v.d.K.). Sometimes two consecutive sections were included on one glass slide. In those cases, only one of the two sections was annotated and the other excluded from further analysis.

#### Sentinel lymph node

Sentinel lymph node slides were digitized using a 3DHistech Pannoramic 250 Flash II slide scanner. Slides were digitized using a 20× objective (resultant pixel resolution of 0.24 microns). After digitization the slides were annotated using the Aperio ImageScope software using a freehand drawing tool. Annotation was performed by a lab technician (M.H.). Annotations were subsequently checked by an experienced pathologist for correctness and completeness (P.B.).

The extra testing set was not annotated, only the pathologist’s report was available for this set.

### Pre-processing steps

The annotations were used to generate binary mask images at the same resolution as the original slides. Any pixel inside an annotated region was labeled as cancer (label 1), whereas all other regions were left blank (label 0).

In addition to the binary annotation mask, we also generated binary tissue masks to separate background from tissue. To this end we performed a simple thresholding procedure on the optical density of the RGB channels. Optical density of a channel is obtained through:


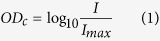


Here *OD*_*c*_ is the optical density of the channel c (Red, Green or Blue), *I* is the intensity of the channel and *I*_*max*_ is the maximum intensity, which is 255 due to 8-bit quantization. By thresholding the optical densities at 0.2, all background could be removed resulting in a binary mask where tissue is labeled 1 and background is labeled 0.

### Convolutional neural network training and application

To train the convolutional neural network we made use of the open-source ‘deep learning’ libraries Theano 0.7 and pylearn2 0.1^25^,^26^.

As it is impossible to feed entire whole-slide images to the network at once, we randomly extracted small patches from the whole-slide image for training. Whole-slide results can then be obtained by applying the network to every pixel in the image.

Patch size in pixels was determined empirically during initial experiments. We tried 64 × 64, 128 × 128 and 256 × 256 pixel patches. The 64 × 64 sized patches performed substantially worse on patch-based accuracy and 256 × 256 sized patches limited convolutional networks depth due to memory limitations of the GPU. As such, we settled on a patch size of 128 × 128.

To allow the network to learn the appearance of cancer (prostate or metastasized breast cancer in this paper), it is important that the small patches contain enough information to allow discrimination between patches with and without cancer. In contrast, when one selects patches which are too large, it is harder for the network to identify the relevant discriminatory features. As such, the correct resolution at which to extract the information had to be determined.

For the prostate cancer experiment, guidelines suggest that initial inspection of biopsy specimens should be performed using a microscope with a 5× objective, already indicating that this is a suitable resolution. Subsequently, we confirmed this in a small observer experiment where an untrained researcher was able to separate cancer and non-cancer patches at 5× (equivalent resolution 0.60 microns per pixel). As such, for this experiment patches were extracted at 5× objective magnification. For the sentinel lymph node slides a similar strategy was used, and here a 10× objective magnification was selected (0.48 microns per pixel).

Patches of 128 × 128 pixels were extracted at the determined resolution level from both cancerous regions and non-cancerous tissue regions using the masks obtained in the pre-processing stage. Extraction was performed such that an equal number of patches for both classes was obtained. During patch extraction, patches were rotated (0, 90, 180 and 270 degrees) and subsequently flipped to make sure the network would not learn rotation-dependent features. This resulted in eight variations of each single patch and was performed for both positive and negative samples. In total this resulted in a total of 920,000 patches for the prostate cancer experiment and 2.6 million patches for the lymph node experiment. Patch-extraction was performed for both the training and the validation sets in both experiments. Test sets in both experiments were only used for final evaluation and were untouched during the training procedure.

After patch extraction, a convolutional neural network was trained where the performance of the network was monitored by assessing the miss-classification rate on the validation set patches. Training was stopped when validation set error did not improve for five epochs. Network structure (e.g. number of layers, filters per layer, number of nodes in fully connected layers) and parameters (e.g. learning rate, momentum) were continuously tweaked to obtain maximum performance on the validation set. In the end, very similar network structures and parameter settings were obtained for both experiments. Training time per epoch was around 80 and 200 minutes per epoch for the prostate cancer and lymph node experiments respectively (GeForce GTX970). Optimal performance was reached after 5 and 12 epochs respectively. The full network specifications can be found in the supplementary files.

After network training, the CNN was converted to a fully convolutional network to allow fast application to the whole slide image^27^. Applying this fully convolutional network to the whole slide image resulted in a likelihood map where each pixel has a continuous likelihood between 0 and 1 of containing cancer. The likelihood map generation time of a prostate biopsy slide is between 5–10 minutes and that of a lymph node slide between 30–40 minutes. However, the average time to analyze a slide is highly dependent on the amount of tissue on the slide. Due to the nature of the procedures, prostate biopsies often contained much less tissue and thus were quicker to process.

Due to the nature of the sentinel lymph node slides, where often only a tiny fraction of the slide contains cancer and most of the slide is covered by lymphocytes, certain normal regions which look more similar to cancer are typically underrepresented in the training data. In other words, the network is not capable of correctly identifying these areas as normal. To tackle this problem, we choose to use a boosting approach similar to the one used by Ciresan *et al*.^9^ We used the initial likelihood maps obtained for the training data set to sample new patches for both the cancerous and non-cancerous class. However, this time we increased the likelihood of adding a patch to the training data if the center pixel of the patch was initially classified wrongly by the network. This process results in additional training data which contains more difficult samples. Subsequently, we re-trained the network on the old and enriched patches and obtained the final likelihood maps for the sentinel lymph node slides.

This boosting procedure was not applied to the prostate cancer detection experiment, as in biopsy slides the area covered by cancer and the area covered by normal tissue is more balanced.

### Evaluation

#### Prostate cancer

A normalized cumulative histogram is calculated on the final whole-slide likelihood image. The histogram was constructed with 100 bins, equally spaced between 0 and 1. In cases where there is no cancer in the slide, the histogram will rise quickly, whereas in cancer cases, the histogram will rise more slowly. To move from this histogram to a slide level label, we used percentile analysis. A percentile is selected and then we inspect at which likelihood we reach this percentile. By doing this for all cases and the performing receiver-operating characteristic (ROC) analysis given this likelihood, we can assess CNN performance at the slide level. We used the validation data set to obtain the optimal percentiles with a step size of 10 for both overall area under the ROC curve and highest specificity at 0.999 sensitivity, which were the median and the 90^th^-percentile respectively.

To assess the confidence interval of the area under the ROC curve, we used bootstrapping using 10,000 bootstrap samples^18^.

#### Sentinel lymph node

The final likelihood map for the sentinel lymph node slides is first thresholded at a likelihood of 0.3 to get rid of all the low likelihood false positives. Subsequently, we performed connected component analysis to obtain all the detected lesions. All components with a diameter smaller than 0.02 mm (10% of the minimum diameter for micro-metastases) are subsequently removed to get rid of spurious detection caused by artifacts (e.g. dust, tissue deformation). To obtain a likelihood per component, the median likelihood across all pixels within each component is calculated.

Given the component segmentations and likelihoods, we perform free-receiver operating characteristic (FROC) at the metastasis level and ROC analysis at the slide level on the test set. On the consecutive set we only performed ROC analysis as we did not have annotations for this set. In FROC analysis we assess for each individual metastasis, being macro-metastases, micro-metastases or ITC, whether it is detected. A metastasis is counted as detected when a component segmentation has a Dice coefficient of at least 0.5 with the annotation. All components which do not coincide with a metastasis are considered false positive detections. The component likelihood is subsequently used to generate the FROC curve. The sensitivity in the FROC curve is expressed with respect to false positive detections in metastasis-free slides.

For ROC analysis, the component with the highest likelihood is used as the slide level score, as this is the component that is actionable. These slide likelihoods were subsequently used to construct the ROC curves at the slide level.

All analysis for the sentinel lymph node cases were performed twice: once assuming ITCs are not considered metastases (i.e. they are ignored) and once assuming ITCs are metastases and as such have to be detected.

## Additional Information

**How to cite this article**: Litjens, G. *et al*. Deep learning as a tool for increased accuracy and efficiency of histopathological diagnosis. *Sci. Rep.* **6**, 26286; doi: 10.1038/srep26286 (2016).

## Supplementary Material

Supplementary Information

Supplementary Information

## Figures and Tables

**Figure 1:**
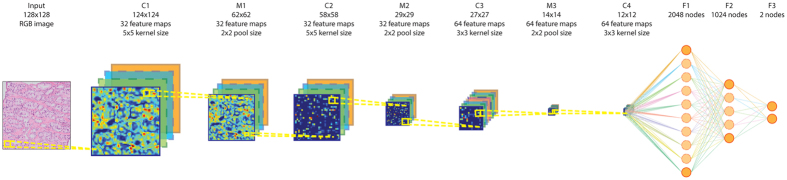
Processing pipeline of a convolutional neural network for the detection of prostate cancer in H&E-stained whole slide biopsy specimens. The four layers indicated with C, meaning a convolutional layer, can be considered a ‘feature extraction’-stage were consecutively higher level features are extracted from the image patch. The layers indicated by the letter M are max pooling layers which reduce image size and provide improved translational invariance to the network. The last three layers are the ‘classification’ layers (indicated with F) which, based on the given features, indicates whether the image patch contains cancer or not. Such a network can subsequently be applied to every pixel in a whole slide image in a ‘sliding window’-fashion^27^.

**Table 1:** Data details for the whole slide biopsy specimens used for the prostate cancer experiments.

Nr. of slides per category	Training	Validation	Test	Total
Cancer	48 (62.94 ± 29.23)	31 (62.32 ± 27.88)	45 (64.90 ± 25.22)	124 (64.02 ± 26.78)
2 + 3	0	1	0	1
3 + 2	0	2	0	2
3 + 3	11	9	14	34
3 + 4	23	9	12	44
3 + 5	0	0	1	1
4 + 3	7	6	10	23
4 + 4	5	1	3	9
4 + 5	2	2	3	7
5 + 3	0	1	0	1
5 + 4	0	0	2	2
Normal	52	19	30	101
Total	100	50	75	225

The first column indicates the categories and the first row indicates the different data sets. For the cancer category, slide distribution is also indicated according to Gleason Score. The numbers between brackets for the ‘Cancer’-row indicate the average volume percentage of cancer within the slides and the corresponding standard deviation.

**Table 2:** Data details for the whole slide sentinel lymph node specimens used for the breast cancer metastasis experiments.

Nr. of slides per category	Training	Validation	Test	Consecutive	Total
*At least one macro-metastasis*	18	5	7	16	**46**
*No macro-metastasis, at least one micro-metastasis*	29	8	8	4	**49**
*No macro- or micro-metastases, at least one instance of ITC*	1	0	1	22	**24**
*No macro- or micro- metastases and no instances of ITC*	50	20	26	56	**152**
*Total*	*98*	*33*	*42*	*98*	*271*

The first column indicates the categories and the first row indicates the different data sets. (ITC = isolated tumor cells).

**Table 3:** Free-response receiver operating characteristic (FROC) and receiver operating characteristic (ROC) analysis in the sentinel lymph node experiment.

FROC analysis	1 FP	2 FP
Sensitivity (incl. ITC)	0.71 (0.39–0.93)	0.74 (0.59–0.94)
Sensitivity (excl. ITC)	0.90 (0.63–0.99)	0.93 (0.78–1.0)
**ROC analysis**	**Area under the curve**	**Specificity at 99.9% sensitivity**
Test (incl. ITC)	0.88 (0.77–0.97)	0.39 (0.33–0.90)
Test (excl. ITC)	0.90 (0.79–0.98)	0.39 (0.32–0.94)
consecutive (incl. ITC)	0.74 (0.65–0.82)	0.02 (0.01–0.30)
consecutive (excl. ITC)	0.88 (0.81–0.93)	0.44 (0.43–0.69)

Mean bootstrap values are given for sensitivity (FROC analysis), area under the curve (ROC analysis) and specificity at 99.9% sensitivity (ROC analysis). 95^th^-percentile confidence intervals obtained through bootstrapping are shown between brackets. (FP = False positive detections per tumor-negative image).
